# Early Cognitive Predictors of 9-Year-Old Spoken Language in Children With Mild to Severe Hearing Loss Using Hearing Aids

**DOI:** 10.3389/fpsyg.2019.02180

**Published:** 2019-09-26

**Authors:** Teresa Y. C. Ching, Linda Cupples, Vivienne Marnane

**Affiliations:** ^1^National Acoustic Laboratories, Sydney, NSW, Australia; ^2^The Hearing CRC, Melbourne, VIC, Australia; ^3^Department of Linguistics, Macquarie University, Sydney, NSW, Australia; ^4^Centre for Language Sciences, Macquarie University, Sydney, NSW, Australia

**Keywords:** short-term memory, language, cognitive predictors, hearing aids, children with hearing loss

## Abstract

This study examined the extent to which cognitive ability at 5 years of age predicted language development from 5 to 9 years of age in a population-based sample of children with hearing loss who participated in the Longitudinal Outcomes of Children with Hearing Impairment (LOCHI) study. The developmental outcomes of 81 children with hearing loss were evaluated at 5 and 9 years of age. Hearing loss ranged from mild to severe degrees, and all participants used hearing aids. They all used spoken language as the primary mode of communication and education. Nine-year-old language was assessed using the Clinical Evaluation of Language Fundamentals – 4th edition (CELF-4), the Peabody Picture Vocabulary Test – 4th edition (PPVT-4), and the Expressive Vocabulary Test – 2nd edition (EVT-2). Multiple regression analyses were conducted to examine the extent to which children’s scores on these standardized assessments were predicted by their cognitive ability (non-verbal IQ and verbal working memory) measured at 5 years of age. The influence of early language scores at 5 years and a range of demographic characteristics on language scores at 9 years of age was evaluated. We found that 5-year-old digit span score was a significant predictor of receptive and expressive language, but not receptive or expressive vocabulary, at 9 years of age. Also, 5-year-old non-word repetition test score was a significant predictor of only expressive language and vocabulary, but not receptive language or vocabulary at 9 years of age. After allowing for the effects of non-verbal IQ and 5-year-old receptive vocabulary, early digit span score (but not non-word repetition score) was a significant predictor of expressive and receptive language scores at 9 years of age. The findings shed light on the unique role of early verbal working memory in predicting the development of receptive and expressive language skills and vocabulary skills in children who use hearing aids.

## Introduction

Children with hearing loss achieve lower language outcomes, on average, than children with normal hearing. Findings from a recent, population-based study, the Longitudinal Outcomes of Children with Hearing Impairment or “LOCHI” study, show that 5-year-old children with hearing loss are about 0.5–1 SD on average behind their normally hearing peers on standardized tests of receptive and expressive language and receptive vocabulary (Cupples et al., 2018). Average scores conceal marked variability, however, in the outcomes achieved by individual children with hearing loss. The demographic and audiological variables that contribute to this variation have been widely studied in recent research, but questions remain regarding the possible influence of early cognitive variables. The current research aimed to shed light on this unresolved issue. In doing so, the intention was not to evaluate the reciprocal view, that early language impacts later cognitive ability, because that view has not been cast into doubt on the basis of recent research (e.g., Botting et al., 2017; Jones et al., 2019). On the other hand, neither Botting et al. (2017) nor Jones et al. (2019) found evidence for an influence of cognitive ability on language outcomes, an important finding that warrants further attention.

### Cognition and Language in Cochlear Implant Users

Much of the past research on this topic has focused on the association between executive function and language outcomes achieved by cochlear implant users. This focus has often been underpinned by a theoretical perspective in which early exposure to sound is seen as vital for the typical development of cognitive abilities that relate to the representation and processing of sequential information, which in turn is associated with language development (Conway et al., 2009, 2011a,b; Pisoni et al., 2016). Although there have been several failures to replicate critical empirical findings cited in support of the theory (e.g., Giustolisi and Emmorey, 2018; Hall et al., 2018; von Koss Torkildsen et al., 2018), evidence for a link between cognitive ability and language outcomes remains.

In a study of 64 cochlear implant users and 74 normally hearing peers, ages 7–27 years, Kronenberger et al. (2014) examined the association between four composite measures of executive function (verbal working memory, spatial working memory, fluency speed skills, and inhibition-concentration) and a composite language measure, which encompassed receptive vocabulary (the Peabody Picture Vocabulary Test – 4th edition; Dunn and Dunn, 2007) and the core language score from the Clinical Evaluation of Language Fundamentals – 4th edition (CELF-4; Semel et al., 2003). A regression analysis, conducted across the two groups of participants combined, showed that non-verbal ability, hearing status, verbal working memory, and spatial working memory each accounted for significant unique variance in language outcomes measured concurrently. However, the strength of the relationship between language and working memory varied according to hearing status, such that verbal working memory was a stronger predictor of language outcomes in cochlear implant users than their normally hearing peers, and spatial working memory was a relatively weaker predictor in cochlear implant users.

In accordance with the need for longitudinal studies to address questions of development, Pisoni et al. (2011) examined the extent to which later language outcomes (at 15–18 years of age) could be predicted by earlier digit span and verbal rehearsal speed (at 8–9 years of age) in a sample of 112 cochlear implant users. The results showed that digit span standard scores were significantly below that of a normative age-matched sample at both assessment time points, and that digit span forward, digit span backward, and verbal rehearsal speed were all significantly positively correlated with later language scores on the PPVT and CELF. The researchers noted further that similar correlations were obtained even when controlling for a range of relevant audiological variables including age at cochlear implantation. An obvious question, however, is whether early cognitive variables would still be associated with later language variables if early language variables were controlled.

### Early Language and Later Language in Cochlear Implant Users

Several recent studies of cochlear implant users have provided evidence that early language abilities are a good predictor of later language and cognitive outcomes. Castellanos et al. (2016a) examined the association between early receptive vocabulary (assessed using the PPVT-3 at 3–6 years of age) and later receptive vocabulary (using the PPVT-4 at 7.8–23.4 years of age) in a small sample of 19 cochlear implant users. They reported a significant positive association between the two vocabulary measures, and noted that, in a regression analysis, demographic and audiological variables explained little additional unique variance in later PPVT-4 once early PPVT-3 was included. Similarly, a study of 51 cochlear implant users reported by Nittrouer et al. (2016) revealed that morpho-syntactic ability at 8.6 years of age, which comprised a set of narrative measures including mean length of utterance (MLU) in morphemes, number of conjunctions, and number of pronouns, was best predicted by the same narrative measures collected at 3 and 6 years of age, and by expressive vocabulary measured at 4 years of age. Castellanos et al. (2016b) found that a parent-reported measure of expressive vocabulary collected within 2.5 years of cochlear implantation strongly predicted long-term language (assessed using PPVT-4 and CELF-4) and memory outcomes (assessed using digit span forward and backward, and visual digit span) from 5 to 16 years later in a sample of 32 cochlear implant users (9–22 years of age at follow-up). Collectively, these results underscore the importance of controlling for early language ability when examining the association between early cognitive predictors and later language outcomes.

Further supporting evidence comes from Hunter et al. (2017) who investigated whether early language, assessed within 1 year of cochlear implantation, could predict later outcomes in language and executive functioning, in particular, verbal working memory. A sample of 36 adolescent and young adult cochlear implant users (ages 11.6–27.4 years) completed assessments of executive functioning (including verbal and visuo-spatial working memory), receptive and expressive language (CELF-4 core language subtests), and receptive vocabulary (PPVT-4). Regression analyses showed that measures of early speech and language ability accounted for significant unique variance in later language outcomes, while controlling for variation in age at implantation (which was also significant), degree of hearing loss, household income, and non-verbal IQ (which were not significant). A total of 46% of variance in later language outcomes was explained. Using the same set of predictor variables, however, a total of 71% of variance in later verbal working memory was explained, with all predictor variables accounting for significant unique variance. Hunter et al. (2017) findings are therefore indicative of an association between early language and later cognitive outcomes, a view that gains further support from the literature via empirical studies examining outcomes in groups of deaf and hard-of-hearing (DHH) people more broadly, rather than cochlear implant users in particular.

### Cognition and Language in Diverse Groups of DHH Individuals

Figueras et al. (2008) reported evidence to suggest that hearing-related group differences in executive function were underpinned by differences in language ability. They found a significant positive association between language ability (receptive vocabulary and sentence comprehension) and executive function (planning, set shifting, working memory, impulse regulation, and visual attention) in both normally hearing children, after controlling for age, and children with hearing loss, after controlling for age, degree of hearing loss, and duration of use of the current device (22 children used cochlear implants and 25 used hearing aids). They also reported, however, that hearing-related group differences in measures of executive function were non-significant once language ability was controlled.

In a similar vein, as noted earlier, both Botting et al. (2017) and Jones et al. (2019) reported evidence to suggest that language ability predicts executive function in children with hearing loss, but executive function does not, in turn, predict language outcomes. Botting et al. (2017) conducted a mediation analysis using concurrent measures of language and executive function, and showed that language ability mediated differences in executive function between groups of deaf and hearing children. More specifically, after controlling for language, group no longer predicted executive function; whereas after controlling for executive function, group differences in language remained significant. On the other hand, Jones et al. (2019) used longitudinal data to demonstrate that early expressive vocabulary (assessed at around 8 years of age) predicted later executive function (at around 10 years old), but not the reverse.

In comparing the results of the studies by Botting et al. (2017) and Jones et al. (2019) with the findings from studies of cochlear implant users, two important methodological differences are noteworthy. As already mentioned, participants in the studies by Botting et al. (2017) and Jones et al. (2019) constituted a more diverse group of children using hearing aids or cochlear implants and various modes of communication, including British Sign Language, Sign supported English, or spoken English. In addition, language was assessed using a single measure of *expressive* vocabulary and executive function using a battery of explicitly *non-verbal* measures. By contrast, assessments of cochlear implant users often targeted *receptive* vocabulary and *verbal* measures of cognitive ability. It is unknown the extent to which these differences in participant samples and methodology might have influenced the findings, but what is apparent is the need for further systematic investigation into whether early measures of cognitive ability predict later language outcomes in children with mild to severe hearing loss who use hearing aids.

### The Current Study

The current study addressed this gap in the literature. The participant sample was drawn from the cohort taking part in the population-based LOCHI study in which children with hearing aids outnumber those with cochlear implants approximately 2:1. Although the advent of universal newborn hearing screening has made it possible for early detection and fitting of hearing devices to children with permanent childhood hearing loss, it remains uncertain as to whether those at risk for suboptimal long-term outcomes for speech and language may be identified through early measures of speech, language, and working memory. We took advantage of the prospective nature of the LOCHI study to examine the influence of early cognitive and language abilities on later language abilities for children with permanent hearing loss using hearing aids. The study measured outcomes of a population-based cohort of about 450 children in Australia who were born with hearing loss and received hearing intervention before 3 years of age. Details of the study have been reported in Ching et al. (2013). As part of the study, the demographic characteristics and developmental outcomes of children were examined at chronological ages of 3, 5, and 9 years of age. The current study draws on data collected at 5 years of age for predicting outcomes collected at 9 years of age.

From a theoretical perspective, we drew on the multi-component model of working memory described by Baddeley et al. (1998) and Baddeley and Hitch (2019), with a particular focus on the role of the phonological short-term store or phonological loop in language learning. According to Baddeley et al. (1998), the phonological loop mediates language learning, especially vocabulary development, by enabling the temporary storage of new phonological forms while long-term representations are established. They also acknowledge, however, that as knowledge of language increases, learners can use that language knowledge to support new word learning and thereby reduce reliance on the phonological loop. In accordance with this view, an evaluation of the role of early phonological memory in later language development should include a control for the impact of early language ability.

In the current study, the capacity of the phonological loop was measured using both memory span for digits and non-word repetition (the ability to repeat an unfamiliar spoken form). These measures were selected in light of positive results from previous research with normally hearing children (e.g., Gathercole et al., 1992; Avons et al., 1998). Language was assessed using standardized measures of receptive and expressive skills, including two measures of vocabulary development. Finally, a measure of non-verbal cognitive ability was included along with other relevant demographic variables (age at hearing aid fitting, degree of hearing loss, and maternal education) to evaluate the unique contribution of our primary predictors to language outcomes.

The research questions addressed in the study were as follows:

1. Does the capacity of the phonological loop, assessed at 5 years of age, predict 9-year-old outcomes in receptive and expressive language and vocabulary in a population-based sample of hearing aid users after controlling for variation in non-verbal cognitive ability and relevant demographic variables? If so, which measure of 5-year-old phonological short-term memory, digit span, or non-word repetition, is more strongly associated with 9-year-old outcomes?
2. Does the capacity of the phonological loop, assessed at 5 years of age, predict 9-year-old outcomes in receptive and expressive language and vocabulary in a population-based sample of hearing aid users after controlling for 5-year-old receptive vocabulary, non-verbal cognitive ability, and relevant demographic variables? If so, which measure of 5-year-old short-term memory, digit span, or non-word repetition, is more strongly associated with 9-year-old outcomes?
  In accordance with the working memory theoretical framework and empirical findings from previous literature with cochlear implant users (e.g., Pisoni et al., 2011), we hypothesized that (1) higher digit span and non-word repetition scores at 5 years of age would be associated with better language and vocabulary outcomes at 9 years of age after controlling for 5-year-old non-verbal ability and relevant demographic variables; and that non-word repetition would be a stronger predictor than digit span (Baddeley et al., 1998). We also hypothesized that (2) these associations would remain significant after allowing for the influence of early receptive vocabulary in addition to non-verbal ability and relevant demographic variables.

## Materials and Methods

### Participants

The Australian Hearing Human Research Ethics Committee approved the protocols used in the current study. Participants in the LOCHI study were included if they continued to use hearing aids by 9 years of age, and completed direct assessments of cognitive and spoken language abilities at 5 and 9 years. Data on measures of 81 participants in the LOCHI study were included in this report. Table 1 provides descriptive statistics of the demographic characteristics of the current sample.

**TABLE 1:** Demographic characteristics of study participants.

**Variable**	**Number of participants (%)**	
Gender (male)	37	(45.7)
**Degree of hearing loss (4FA HL)**		
Mild (≤40 dB)	29	(35.8)
Moderate (41–60 dB)	40	(49.4)
Severe (61–80 dB)	12	(14.8)
**Maternal education (*n* = 144)**		
1. University qualification	34	(42.0)
2. Diploma or certificate	22	(27.2)
3. 12 years or less of schooling	25	(30.9)
**Age at first fitting of hearing aids (months)**		
Mean (SD)	7.8 (8.2)	
Median	3.8	
75th percentile	10.1	

*4FA HL, the average of hearing threshold levels at 0.5, 1, 2, and 4 KHz in the better ear.*

### Procedure

Parents of participants provided written informed consent to the protocol approved by the local institutional human research review board. As part of the LOCHI study, each child was assessed directly by research speech pathologists on norm-referenced tests using standard protocols when they turned 5 and 9 years of age. All data were audited and checked for reliability by double scoring 10% of the evaluations.

### Measures

The 5-year-old assessment battery included the PPVT-4 (Dunn and Dunn, 2007), the Memory for Digits (MD) subtest of the Comprehensive Test of Phonological Processing (CTOPP; Wagner et al., 1999), the non-word repetition test (NRT) (Dollaghan and Campbell, 1998), and the Wechsler Non-verbal Scale of Ability (WNV; Wechsler and Naglieri, 2006). The 9-year-old assessment battery included the CELF-4 (Semel et al., 2003), the PPVT-4, and the Expressive Vocabulary Test (EVT; Williams, 2007).

The PPVT-4 is a standardized test of receptive vocabulary, using a four-alternative-forced choice, picture-pointing format in administration. It gives an overall score on receptive vocabulary.

The EVT is a standardized test of expressive vocabulary. It gives an overall score on expressive vocabulary.

The MD subtest of the CTOPP is a standardized test of capacity of the phonological loop. Recorded digits are presented at a rate of two per second, and forward-only recall is measured. It gives an overall score on phonological short-term memory.

The NRT is another measure of the phonological loop (Gathercole and Baddeley, 1990) in short-term memory. Recorded non-words are presented at a comfortable listening level and the participant is required to repeat back the non-words heard. Responses are recorded and transcribed phonetically for scoring of the number of vowels and consonants correctly repeated. It gives an overall score on phonological short-term memory in terms of phoneme correct score.

The WNV is a standardized test of non-verbal cognitive ability. It gives a full-scale IQ score.

The CELF is a standardized test of spoken English. The test includes verbal tasks which enable children to demonstrate understanding of and ability to produce English language structures. It gives an overall core language score, and two subtest scores – receptive language and expressive language. It also gives a language memory score.

Parents were requested to complete a custom-designed questionnaire to provide demographic information. Audiological information was retrieved from individual clinical files, with permission from parents. All hearing level information and hearing device information were current within 6 months of the evaluation, and at a time closest to the actual evaluation date for each child.

### Statistical Analyses

Descriptive statistics were used to report quantitative outcomes for each measure. To examine the relations between early measures of language and working memory at 5 years of age and later language outcomes, correlational analyses were carried out. To determine whether any relations found between early working memory remained after accounting for the effects of early language abilities and other demographic variables and non-verbal intelligence, multiple linear regression analyses were conducted. Two models were fitted with the 9-year CELF Receptive Language and Expressive Language standard scores as dependent variables with repeated measures. In the first model, age at hearing aid fitting, maternal educational level (three categories: university vs. certificate or diploma vs. schooling of 12 years or less), degree of hearing loss [averaged hearing levels at 0.5, 1, 2, and 4 kHz, 4FA in decibel hearing level (4FA dB HL)], and 5-year-old standard scores for WNV, MD, and NRT were used as independent variables to predict 9-year-old language outcomes. The second model included all predictor variables together with 5-year-old PPVT scores to examine the effects of early cognitive measures after allowing for the effect of early language ability. To investigate the relationship between early measures and later vocabulary outcomes, two separate models were fitted in the same manner, but using 9-year-old PPVT scores and EVT scores as dependent variables. Statistical significance was set at the 0.05 level.

## Results

Demographic characteristics of participants in this study are reported in Table 1.

Descriptive statistics for the scores of each of the outcome measures are shown in Table 2. The mean scores on the PPVT-4, EVT, and the CELF-4 measures of receptive and expressive language were within 1 SD (15) of the norm-referenced mean score of 100. The mean scores on the CTOPP Memories for Digit test is within 1 SD (3) of the norm-referenced mean score of 10.

**TABLE 2:** Descriptive statistics for language and cognitive measures.

	**Measures at 5 years of age**		**Measures at 9 years of age**	
	**Mean (*SD*)**	**Range**	**Mean (*SD*)**	**Range**
Y5PPVT	93.7 (14.7)	58–134		
Y5WNV	106.6 (14.3)	58–132		
Y5MD	9.1 (3.1)	1–16		
Y5NRT	57.4 (13.3)	22–86		
Y9RecLg			86.6 (14.9)	50–112
Y9ExpLg			88.8 (18.5)	49–118
Y9PPVT			94.0 (14.7)	61–134
Y9EVT			95.1 (12.2)	69–125
Y9WNV			101.1 (16.9)	43–132

*Y5PPVT, PPVT-4 Receptive Vocabulary standard score at 5 years of age; Y5WNV, Wechsler Non-Verbal Full Scale IQ at 5 years of age; Y5MD, CTOPP Memory for digits subtest standard score at 5 years of age; Y5NRT, non-word repetition test phoneme correct score at 5 years of age; Y9RecLg, CELF Receptive Language standard score at 9 years of age (*n* = 78); Y9ExpLg, CELF Expressive Language standard score at 9 years of age (*n* = 78); Y9PPVT, PPVT-4 Receptive Vocabulary standard score at 9 years of age; Y9EVT, EVT Expressive Vocabulary standard score at 9 years of age (*n* = 80), and Y9WNV, Wechsler Non-Verbal Full Scale IQ at 9 years of age (*n* = 78).*

Correlations between language scores and non-verbal IQ at 9 years of age and characteristics at 5 years of age are shown in Table 3. Early receptive vocabulary scores, non-verbal IQ, digit span, and non-word repetition scores were significantly correlated with receptive and expressive language and vocabulary scores at 9 years of age. There were no significant associations between language performance at 9 years of age and age at first fitting of hearing aids, or degree of hearing loss of the children. There was no significant relation between maternal education at 5 years of age and non-verbal cognitive ability at 9 years of age.

**TABLE 3:** Correlations (Pearson’s *r*) between demographic and early predictors and long-term language outcomes.

	**9-Year-old outcomes**				
	**Y9RecLg**	**Y9ExpLg**	**Y9PPVT**	**Y9EVT**	**Y9WNV**
	**(*n* = 78)**	**(*n* = 78)**	**(*n* = 80)**	**(*n* = 80)**	**(*n* = 78)**
AgeHA	0.04	–0.07	0.07	–0.01	0.05
BE4FA	–0.14	–0.08	–0.09	–0.13	0.01
Y5MEdn	−0.38^∗^	–0.42^∗∗^	−0.36^∗^	–0.43^∗∗^	0.15
Y5PPVT	0.62^∗∗^	0.72^∗∗^	0.72^∗∗^	0.72^∗∗^	0.31^∗^
Y5WNV	0.59^∗∗^	0.55^∗∗^	0.43^∗∗^	0.56^∗∗^	0.65^∗∗^
Y5MD	0.54^∗∗^	0.58^∗∗^	0.42^∗∗^	0.39^∗^	0.31^∗^
Y5NRT	0.37^∗^	0.51^∗∗^	0.37^∗^	0.43^∗∗^	0.21

*AgeHA, Age at Hearing Aid Fitting; BE4FA, Four Frequency Average Hearing Loss in the better ear; Y5MatEd, Maternal Education (1 = university; 2 = diploma/certificate; 3 = ≤12 years formal schooling) at 5 years of age; Y5PPVT, PPVT-4 Receptive Vocabulary standard score at 5 years of age; Y5WNV, Wechsler Non-Verbal Full Scale IQ at 5 years of age; Y5MD, CTOPP Memory for digits subtest standard score at 5 years of age; Y5NRT, non-word repetition test phoneme correct score at 5 years of age; Y9RecLg, CELF Receptive Language standard score at 9 years of age; Y9ExpLg, CELF Expressive Language standard score at 9 years of age; Y9PPVT, PPVT-4 Receptive Vocabulary standard score at 9 years of age; Y9EVT, EVT Expressive Vocabulary standard score at 9 years of age; and Y9WNV, Wechsler Non-Verbal Full Scale IQ at 9 years of age. ^∗^*p* < 0.01, ^∗∗^*p* < 0.001.*

Regression models predicting language and vocabulary at 9 years of age are shown in Table 4. Non-verbal cognitive ability accounted for significant variance in language and vocabulary abilities at 9 years of age. Phonological memory as measured by a digit span test accounted for significant variance in 9-year-old language scores, but not vocabulary scores. The NRT score was a significant predictor of expressive language and vocabulary, but not receptive language and vocabulary at 9 years of age. For both 9-year-old language and vocabulary measures, adding early receptive vocabulary score at 5 years of age resulted in a significant increase in the variance accounted for by the models. After adding the early receptive vocabulary score as a predictor variable, non-word repetition score was no longer a significant predictor of expressive language or vocabulary at 9 years of age. In summary, the full models incorporating early cognitive ability and language measured at 5 years of age together with demographic characteristics accounted for 61% variance in receptive language and 68% in expressive language scores at 9 years of age. Significant predictors included non-verbal IQ at 5 years, phonological short-term memory measured by a digit span test at 5 years, and receptive vocabulary at 5 years of age. The full models also accounted for 55% in receptive vocabulary and 63% in expressive vocabulary at 9 years of age. The only significant predictor for 9-year-old receptive vocabulary was 5-year-old receptive vocabulary. Significant predictors for 9-year-old expressive vocabulary included non-verbal IQ and receptive vocabulary measured at 5 years of age.

**TABLE 4:** Multiple regression summary table showing unstandardized coefficient estimates (*b*-values) and significance levels (*p*-values) of predictor variables for outcomes of children with hearing aids (*p*-values in parentheses).

**Predictor**	**9-Year-old language**				**9-Year-old vocabulary**			
	**Receptive (Y9RecLg)**		**Expressive (Y9ExpLg)**		**Receptive (Y9PPVT)**		**Expressive (Y9EVT)**	
(ln)AgeHA_a_	0.01 (0.89)	0.05 (0.52)	−0.06 (0.42)	−0.02 (0.77)	0.07 (0.48)	0.13 (0.09)	−0.01 (0.87)	0.04 (0.59)
BE4FA	−0.16 (0.05)	−0.13 (0.08)	−0.12 (0.13)	−0.08 (0.22)	−0.06 (0.54)	−0.03 (0.70)	−0.12 (0.17)	−0.10 (0.18)
Y5WNV	**0.43 (<0.001)**	**0.35 (<0.001)**	**0.38 (<0.001)**	**0.28 (<0.001)**	**0.31 (0.004)**	0.17 (0.05)	**0.46 (<0.001)**	**0.33 (<0.001)**
Y5MD	**0.37 (<0.001)**	**0.31 (0.001)**	**0.34 (0.001)**	**0.27 (0.002)**	0.20 (0.12)	0.09 (0.36)	0.05 (0.62)	−0.04 (0.68)
Y5NRT	0.12 (0.20)	0.01 (0.92)	**0.25 (0.008)**	0.11 (0.16)	0.16 (0.19)	−0.02 (0.85)	**0.24 (0.02)**	0.10 (0.27)
Y5MatEdn_b_	−0.003, 0.14 (0.97)	−0.04, 0.14 (0.70)	0.05, 0.07 (0.64)	0.01, 0.08 (0.91)	0.12, 0.03 (0.37)	0.08, 0.03 (0.45)	0.17, 0.03 (0.14)	0.14, 0.03 (0.14)
Y5PPVT		**0.36 (<0.001)**		**0.43 (<0.001)**		**0.62 (<0.001)**		**0.53 (<0.001)**
Sample size	78	78	78	78	80	80	80	80
Adjusted *R*^2^	0.52	0.61	0.55	0.68	0.26	0.55	0.42	0.63

*Bolded entries indicate significance at <0.05 level. AgeHA, Age at Hearing Aid Fitting; BE4FA, Four Frequency Average Hearing Loss in the better ear; Y5WNV, Wechsler Non-Verbal Full Scale IQ at 5 years of age; Y5MD, CTOPP Memory for digits subtest standard score at 5 years of age; Y5NRT, non-word repetition test phoneme correct score at 5 years of age; Y5MatEd, Maternal Education (1 = university; 2 = diploma/certificate; 3 = ≤12 years formal schooling) at 5 years of age; Y5PPVT, PPVT-4 Receptive Vocabulary standard score at 5 years of age; Y9RecLg, CELF Receptive Language standard score at 9 years of age; Y9ExpLg, CELF Expressive Language standard score at 9 years of age; Y9PPVT, PPVT-4 Receptive Vocabulary standard score at 9 years of age; Y9EVT, EVT Expressive Vocabulary standard score at 9 years of age. ^*a*^(ln) indicates that AgeHA was transformed using the natural logarithm. ^*b*^For maternal education, the first coefficient estimate is for diploma relative to school, and the second coefficient estimate is for university relative to school. The *p*-value for MatEd is for the overall test of MatEd.*

## Discussion

This study reports findings that extend current knowledge, focusing on the early cognitive predictors of later language abilities in a prospective cohort of children who received early intervention for mild to severe hearing loss using HAs. On average, children’s receptive and expressive language scores were around 1 SD below the normative mean, whereas vocabulary scores were within −0.5 SD of the mean.

To address the first research question of whether the capacity of the phonological loop assessed at 5 years of age predicted 9-year-old language outcomes, we found that higher digit span and non-word repetition scores at 5 years of age were significantly associated with better language and vocabulary skills measured at 9 years of age. Of the early cognitive predictors, 5-year-old digit span score was a significant predictor of receptive and expressive language but not receptive or expressive vocabulary. Further, 5-year-old NRT score was a significant predictor of only expressive language and vocabulary, but not receptive language or vocabulary. We also found that higher maternal education and higher non-verbal ability were significantly associated with higher language and vocabulary scores at 9 years of age. However, the regression analyses revealed that only non-verbal ability accounted for unique variance. The failure to find a unique contribution of maternal education is probably not due to its association with non-verbal IQ because the correlation between them is not significant (*p* > 0.05; Table 3). Regression analyses revealed that non-verbal IQ measured at 5 years of age was a significant predictor of 9-year-old language and vocabulary.

To address the second research question of whether 5-year-old phonological short-term memory predicted 9-year-old language outcomes after controlling for 5-year-old receptive vocabulary, we found that 5-year-old digit span score was a significant predictor of 9-year-old expressive and receptive language score, after allowing for the effects of non-verbal IQ and 5-year-old receptive vocabulary. Non-word repetition was no longer a significant predictor of expressive language or expressive vocabulary after allowing for the effects of non-verbal IQ and 5-year-old receptive vocabulary.

The significant association between phonological short-term memory, measured using forward digit span, and 9-year-old language is consistent with findings reported for cochlear implant users (Kronenberger et al., 2014), and for a combined group of hearing aid and cochlear implant users (Figueras et al., 2008). Whereas these previous studies involved concurrent assessments and used composite measures of verbal working memory and language in analyses, the current study used a longitudinal design and showed that early digit span forward, rather than non-word repetition, significantly predicted later expressive and receptive language skills but not vocabulary skills. Importantly, digit span forward was a significant predictor after allowing for the effect of early receptive vocabulary score. The predictive relationship between early cognitive measures and later language abilities is also consistent with findings in Pisoni et al. (2011) longitudinal study of children and adolescents using cochlear implants.

These findings are also broadly consistent with the theoretical framework described by Baddeley et al. (1998) and Baddeley and Hitch (2019). However, whereas Baddeley et al. (1998) suggested that non-word repetition may be a more sensitive measure of the capacity of the phonological loop than digit span, the current data suggest a stronger role for digit span in predicting later language outcomes. Furthermore, by contrast with Baddeley et al.’s (1998) focus on the particular role of the phonological loop in vocabulary acquisition, the current findings provide stronger evidence that early digit span is associated with later language ability considered more broadly (as assessed using the CELF-4). Despite these relatively minor departures from specific theoretical expectations, we interpret our findings as generally consistent with the working memory model and with the assertion that, for children with mild to severe hearing loss who use hearing aids and communicate using spoken language, the capacity of the phonological loop at 5 years of age appears to play an important role in language development.

The question remains: why was digit span forward a stronger predictor of later language abilities than the NRT score? Non-word repetition is a complex task requiring a child to identify a novel string of heard phonemes, retain this string in phonological short-term memory, and produce the same sequence as speech output. Although both digit span forward and non-word repetition tasks require a child to plan and execute the sequence of articulatory gestures to yield a phonological output that corresponds to a retrieved memory representation, articulatory accuracy has a potentially greater effect on the non-word repetition score than a digit span score because a single phoneme deviation is scored as an error in the former but not the latter (Gathercole and Baddeley, 1996). Many children with hearing loss have impoverished phonological representations as a consequence of their auditory deficits and the distorted signals received through hearing devices (e.g., Kronenberger et al., 2014), and may have phonological production systems that will never be fully accurate. As such, performance in the non-word repetition task may be limited by children’s speech output skills (e.g., Snowling and Hulme, 1989). That said, it is important to note that Avons et al. (1998) also reported a stronger association between early digit span and later vocabulary scores than between non-word repetition and later vocabulary scores in a sample of normally hearing children assessed at 5 and 6 years of age. Furthermore, in that study, articulation rate was not a significant predictor.

A striking aspect of the findings reported here is the strong and significant association between early receptive vocabulary and later expressive and receptive language and vocabulary. When added to the regression models for the four 9-year-old language and vocabulary measures, 5-year-old receptive vocabulary accounted for significant unique variance ranging from 9 to 29% after controlling for all other predictor variables. This result is consistent with findings reported for users of cochlear implants (e.g., Castellanos et al., 2016a, b; Nittrouer et al., 2016; Hunter et al., 2017). It also provides support for the proposed role of language knowledge in supporting further language development, and thereby potentially reducing reliance on the phonological loop as the primary language learning device (Baddeley et al., 1998). Some evidence for a reduction in the role of phonological short-term memory in predicting later language ability comes from our findings for non-word repetition, which was a significant predictor of expressive language and vocabulary after controlling for digit span, non-verbal ability, and relevant demographic variables, but became non-significant with the addition of receptive vocabulary as a predictor.

The observed relation between early cognitive abilities and later expressive and receptive language and vocabulary development implies that early forward digit span may be used as a screening tool for audiologists to identify children who may be at risk of later language difficulties, so that the children can be referred for professional assessment and treatment. The significant association between 5-year-old receptive vocabulary and 9-year-old language abilities supports the use of early language assessments to inform what the targets of language intervention should be. Despite early intervention for the cohort of children with hearing loss reported in this study, Table 2 shows that the mean language scores were within 1 SD below the mean of the normal population, suggesting that some children with hearing loss exhibit language deficits at school age that is potentially avoidable if a digit span test could be used as an early screener to expedite referral for language assessment and intervention. This offers the opportunity to capitalize on the benefits due to early detection and treatment of hearing loss (Ching et al., 2017) by optimizing habitation strategies, including considerations for increase in intensity of early language intervention and considerations for alternative hearing devices, such as cochlear implantation, for those who may be in need.

The findings reported in this study are drawn from a cohort of children born with hearing loss who used spoken language as the primary mode of communication and early education. Therefore, these findings should not be generalized to children who communicate using sign language.

Age at hearing aid fitting did not reach significance level in the regression analyses. This finding may be explained in terms of the restricted range of age at hearing aid fitting for the cohort in this report, and the significant association between age at fitting and 5-year-old language outcomes. The current results were drawn from a sub-sample of the LOCHI cohort who use hearing aids, who completed all the spoken language and cognitive measures at 5 years and 9 years of age, and who used speech to communicate. The sample received very early fitting of hearing aids (median: 3.8 months, upper quartile: 10.1 months). In an earlier report (Ching et al., 2017), we showed that earlier fitting was significantly associated with better language at 5 years of age. In the current investigation, 5-year-old receptive vocabulary and cognitive abilities were included as predictors in the regression analyses. Therefore, it is not surprising that after allowing for the effects of early language and cognitive abilities, age at fitting does not account for unique variance in 9-year-old language and vocabulary outcomes.

Future investigations on predictors of language outcomes of children using hearing aids at 9 years will need to include a wider range of factors reported in the literature (e.g., Tomblin et al., 2015) than was used in this study. For example, we did not include hearing device use or aided audibility in this study partly because the current focus is on the role of early cognitive factors on later language development; and partly because our earlier reports on the LOCHI cohort at 3 and 5 years of age showed that these factors did not account for unique variance after allowing for the effects of a range of child- and family-related factors (Ching et al., 2013, 2018a,b). Even though the question of the link between cognitive abilities and language development is not new, the current study is the first to investigate this relationship in a prospective study of a population-based cohort of children with mild to severe hearing loss using hearing aids.

We conclude that early phonological short-term memory assessed using a digit span task significantly predicted later expressive and receptive language abilities, even after allowing for the effect of early receptive vocabulary. Future studies will examine the importance of phonological working memory in speech perception in children with hearing loss.

## Data Availability Statement

The datasets for this study will not be made publicly available because the ethics approval for this study does not include the dissemination of the dataset.

## Ethics Statement

This study was carried out in accordance with the recommendations of the ethics guidelines of the Australian Hearing Human Research Ethics Committee with written informed consent from all subjects. All subjects gave written informed consent in accordance with the Declaration of Helsinki. The protocol was approved by the Australian Hearing Human Research Ethics Committee.

## Author Contributions

TC conceptualized and designed the study, took overall responsibility for all aspects of the study, and drafted the manuscript. LC consulted in the design of the study, and drafted and reviewed the manuscript. VM coordinated the acquisition of data and collation of data, and reviewed the manuscript. TC, LC, and VM approved the final manuscript as submitted.

## Conflict of Interest

The authors declare that the research was conducted in the absence of any commercial or financial relationships that could be construed as a potential conflict of interest.
